# A Bmp Reporter with Ultrasensitive Characteristics Reveals That High Bmp Signaling Is Not Required for Cortical Hem Fate

**DOI:** 10.1371/journal.pone.0044009

**Published:** 2012-09-11

**Authors:** Linda T. Doan, Anna L. Javier, Nicole M. Furr, Kevin L. Nguyen, Ken W. Cho, Edwin S. Monuki

**Affiliations:** 1 Department of Developmental and Cell Biology, School of Biological Sciences, University of California Irvine, Irvine, California, United States of America; 2 Department of Pathology and Laboratory Medicine, School of Medicine, University of California Irvine, Irvine, California, United States of America; 3 Sue and Bill Gross Stem Cell Research Center, University of California Irvine, Irvine, California, United States of America; Ecole Normale Supérieure de Lyon, France

## Abstract

Insights into Bone morphogenetic protein (Bmp) functions during forebrain development have been limited by a lack of Bmp signaling readouts. Here we used a novel Bmp signaling reporter (“BRE-gal” mice) to study Bmp signaling in the dorsal telencephalon. At early stages, BRE-gal expression was restricted to the dorsal telencephalic midline. At later stages, strong BRE-gal expression occurred in neurons of the marginal zone and dentate gyrus. Comparisons to nuclear phospho-Smad1/5/8 (pSmad) and Msx1 indicated that BRE-gal expression occurred exclusively in neural cells with high-level Bmp signaling. BRE-gal responsiveness to Bmps was confirmed in reporter-negative cortical cells cultured with Bmp4, and both *in vivo* and *in vitro*, BRE-gal expression was switch-like, or ultrasensitive. In the early dorsal telencephalon, BRE-gal expression negatively correlated with the cortical selector gene *Lhx2*, indicating a BRE-gal expression border that coincides with the cortex-hem boundary. However, in Lhx2 null chimeras, neither BRE-gal nor nuclear pSmad increases were observed in ectopic hem cells. These findings establish BRE-gal as an ultrasensitive reporter of Bmp signaling in the dorsal telencephalon, imply that hem fate can be specified at different Bmp signaling intensities, and suggest that Lhx2 primarily regulates the responses to – rather than the intensity of – Bmp signaling in dorsal telencephalic cells.

## Introduction

Bone morphogenetic proteins (Bmps) are intimately involved in many nervous system processes, spanning from the induction of neuroectoderm to the maintenance of adult stem cells. However, Bmps in the developing forebrain have been difficult to study due to the early embryonic lethality of some Bmp loss-of-function mutants and to Bmp redundancy [1]. In addition, specific readouts of Bmp signaling are limited. Bmp target genes, such as *Msx1* [2], [3], have complex promoter-regulatory regions that respond to multiple signaling pathways. Nuclear phosphorylated Smad1/5/8 (hereafter referred to as “pSmad”) is a direct readout of Bmp signaling, but nuclear pSmad has limitations as a quantitative readout and does not report transcriptional activity. To overcome these limitations, other groups have generated Bmp reporter mice using a 47-bp Smad-binding enhancer (SBE) from the mouse *Id1* gene [4], [5], [6]. In these mice, however, reporter expression in the dorsal telencephalon – our system of interest – has not been well characterized or appears weak to absent [4], [5], [6].

The dorsal telencephalon, also referred to as the pallium, gives rise to the cerebral cortex and is a highly patterned and complex region of the forebrain. Neural tube closure at the dorsal telencephalic midline (DTM) generates the roof plate, an important signaling center that produces several Bmps [7]. The roof plate then differentiates into and induces multiple DTM tissues: 1) choroid plaque at the immediate midline, 2) choroid plexus epithelium (CPE) adjacent to the choroid plaque bilaterally, and 3) cortical hem between the CPE and cortical primordium [8], [9], [10]. These DTM tissues develop between the bilateral cortical primordia, which constitute most of the dorsal telencephalon.

Several studies implicate Bmp signaling in the induction of DTM tissues. Bmps expressed in the roof plate set up a nuclear pSmad gradient in the dorsal telencephalon, with highest pSmad levels in the immediate midline and progressively lower levels more laterally [11]. Roof plate ablation, which severely curtails Bmp production, leads to a reduced and flattened nuclear pSmad gradient and associated absences of DTM tissues and gene expression [9], [11], [12]. Similar DTM defects occur in Bmp receptor null mice [13], [14], and exogenous Bmp4 can partially rescue CPE fate in roof plate ablated explants [11].

One DTM tissue that depends on the roof plate and Bmp receptors for its induction is the cortical hem, the transient Bmp- and Wnt-producing signaling center [15] that functions as a hippocampal organizer [16]. In addition to the requirement for high-level Bmp signaling, suppression of the cortical selector gene *Lhx2* has been implicated in hem induction. *Lhx2* is a homeobox gene expressed in the cortical primordium, but not in the hem, where high-level Bmp signaling appears to suppress Lhx2 expression [9], [10]. Constitutive or mosaic Lhx2 loss in neuroepithelial cells results in ectopic hem induction within the medial wall of the cortical primordium (medial pallium or hippocampal anlagen) [10], [16], [17]. Thus, in the medial pallium, where Bmp signaling is normally lower than in the DTM [11], Lhx2 suppresses hem fate. However, whether Lhx2 does so by suppressing Bmp signaling intensity or by regulating how cells respond to Bmp signals has been uncertain.

Here we characterize a novel Bmp activity reporter (“BRE-gal” mice) in the dorsal telencephalon, then use it to address Bmp signaling during the differentiation of cortical hem cells. As described in the companion manuscript, this reporter is based on a 46-bp fragment from the 5′ upstream region of the *Xenopus Id3* gene, which contains a 16-bp Bmp response element (BRE) [18]. This BRE contains binding sites for Smad1 and Smad4 separated by 5 bp, and this precise spacing is required for normal BRE activity [19]. Highly conserved across species, this BRE can robustly and faithfully drive reporter expression in tissues known to require Bmp signaling in transgenic frogs, fruit flies, and zebrafish, including in the brain [18], [19], [20]. In this study, we show that BRE-gal expression in the mouse dorsal telencephalon occurs exclusively in neural cells with high Bmp signaling, where its expression is robust and non-mosaic. BRE-gal expression borders are sharp *in vivo*, and BRE-gal activation by exogenous Bmp4 *in vitro* displays strong ultrasensitivity. We also describe BRE-gal expression in marginal zone and dentate gyrus neurons, and find that neither BRE-gal activity nor high pSmad levels are seen in ectopic hem cells in Lhx2-null chimeras, which suggests that cortical hem differentiation can occur at lower Bmp signaling intensities than those seen in the DTM, if Lhx2 is absent.

## Results

### Generation of BRE-gal Reporter Mice

**Figure 1:**
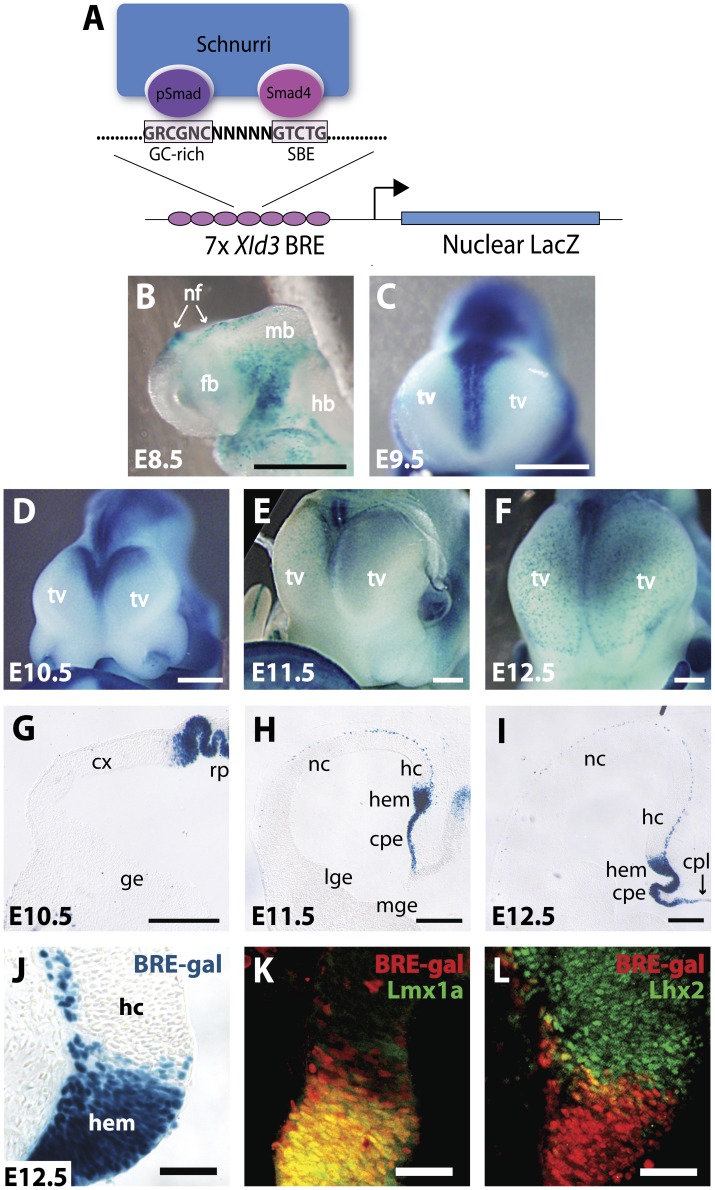
Robust and non-mosaic BRE-gal expression in dorsal telencephalic midline. (**A**) Schematic of the BRE-gal construct showing the 16 bp core of the *XId3* BRE with Smad1 and Smad4 binding sites separated by 5 bp, which allows for docking by the co-factor Schnurri. Seven concatemerized BREs, all in sense orientation, are upstream of the *XId3* minimal promoter and nuclear lacZ coding sequence. (**B–F**) Xgal stains of E8.5-E12.5 whole mount embryos. In the forebrain, BRE-gal expression is initiated in the E8.5 neural folds (nf) (B) and is strongly expressed in the DTM through E12.5 (C–F). Scale bars: 0.5 mm. (**G–I**) Xgal stains of E10.5–E12.5 coronal cryosections. BRE-gal expression is robust and non-mosaic in the DTM (hem and cpe). Scattered labeling is also seen in the E11.5 and E12.5 marginal zone (H,I). Scale bars: 200 um. (**J–L**) Xgal or IHC of E12.5 coronal cryosections. BRE-gal expression coincides with Lmx1a, but not with Lhx2, indicating that the BRE-gal expression border approximates the cortex-hem boundary (CHB). Scale bar: 50 um. Abbr: cpe, choroid plexus epithelium; cpl, choroid plaque; cx, cortex; fb, forebrain; ge, ganglionic eminence; hc, hippocampal primordium; hb, hindbrain; mb, midbrain; nc, neocortical primordium; nf, neural folds; rp, roof plate; SBE, Smad binding element; tv, telencephalic vesicle.

Details on the generation of these mice are provided in the companion manuscript by Javier et al. Briefly, seven copies of a 46-bp *XId3* promoter region containing the BRE were inserted in sense orientation upstream of the *XId3* minimal promoter and nuclear lacZ coding sequence (Fig. 1A). Pronuclear injection yielded six founder lines, but only two lines yielded detectable lacZ expression, which was relatively weak and highly mosaic (data not shown). We then identified a stably-transfected mouse embryonic stem cell (mESC) line with high Bmp4 responsivity *in vitro*, and injected this mESC line into blastocysts to generate two founders that produced progeny with indistinguishable and robust BRE-gal expression *in vivo*. As described in the accompanying manuscript, BRE-gal expression was seen in many, but not all, developing tissues associated with high BMP signaling, similar to BRE-gal transgenic *Xenopus*, *Drosophila* and *Danio* [18], [19], [20].

### BRE-gal Expression in the Dorsal Telencephalon is Restricted to the DTM

Within the developing central nervous system (CNS), BRE-gal expression was strong in the telencephalon, diencephalon, eye, and spinal cord, and these patterns were maintained stably across at least three generations. To characterize BRE-gal expression in the telencephalon, we first analyzed E8.5–12.5 whole mount embryos and sections by Xgal staining. At E8.5, BRE-gal was expressed in the neural folds prior to neural tube closure (Fig. 1B). After closure, BRE-gal was expressed in the DTM throughout the E9.5–E12.5 period (Fig. 1C–I). Expression at E9.5 was restricted to the roof plate, then continued at later stages in tissues derived from or induced by the roof plate (CPE and cortical hem; [9]) (Fig. 1G–I). BRE-gal expression in these DTM tissues was not only strong, but also non-mosaic (i.e. it occurred in all DTM cells) based on the Xgal stains and on confocal images of sections immunolabelled for lacZ (data not shown). However, expression was reduced in the choroid plaque at the immediate midline (Fig. 1I). Interestingly, BRE-gal expression was not detected in non-neural tissues surrounding the forebrain – the mesenchyme and epidermal ectoderm – in which Bmp signaling is known to be relatively high [1], [7]. Thus, BRE-gal expression in the dorsal telencephalon was neural-specific, and occurred in some, but not all, tissues associated with high Bmp signaling. Similar tissue specificities and regional restrictions within the telencephalon were observed in the two pronuclear-injected lines with much weaker and more mosaic expression (data not shown), suggesting that spatial patterns of BRE-gal expression were less integration site-dependent than its expression level or mosaicism.

Because the BRE-gal transgenic construct included the *XId3* minimal promoter, we also compared BRE-gal and Id3 expression. In the embryonic forebrain, Id3 expression was highest in the cortical hem and lower in the medial pallium, CPE, and head mesenchyme, based on Allen Brain Atlas images and our own *in situ* hybridizations (Fig. S2). This is distinct from BRE-gal, which was uniformly expressed in the hem and CPE, and absent from the hippocampal primordium and surrounding mesenchyme. In addition, Id3 and BRE-gal expression differed dramatically in the mature hippocampus (see below). We therefore concluded that neither the BRE nor minimal *XId3* promoter are sufficient to confer Id3-specific expression patterns.

### BRE-gal is Expressed and Maintained in Cortical Marginal Zone Neurons

**Figure 2:**
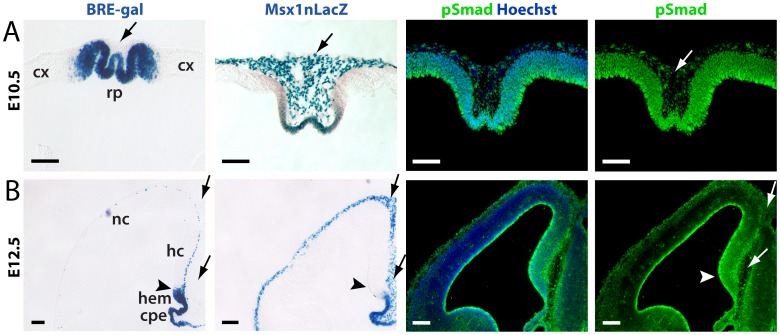
BRE-gal coexpression with other Bmp signaling readouts in the DTM. Xgal or IHC of E10.5 (A) and E12.5 (B) coronal sections. BRE-gal is sharply restricted to the DTM, and excluded from cortex and mesenchyme. pSmad is present in the neuroepithelium (graded from high dorsomedial to low ventrolateral) and in the mesenchyme at both stages. Like pSmad, but in contrast to BRE-gal, Msx1-nlacZ is expressed in both the neuroepithelium and mesenchyme. Within the neuroepithelium, the Msx1-nlacZ expression domain is smaller than the BRE-gal domain at both stages. Scale bars: 100 um. Arrows designate mesenchyme; arrowheads designate the cortex-hem boundary. Abbr: cpe, choroid plexus epithelium; cx, cortex; hc, hippocampal primordium; nc, neocortical primordium; rp, roof plate.

**Figure 3:**
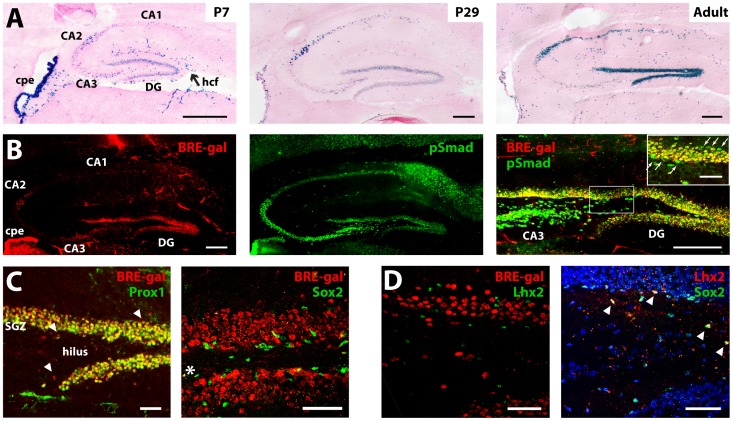
BRE-gal induction in the postnatal hippocampus. (**A**) Xgal stains of P7, P29, and adult (10 months) sagittal sections, with eosin counterstain. Although BRE-gal is absent from the hippocampal anlagen before E17.5 (Figs. 1, S1A), BRE-gal expression becomes detectable in the dentate gyrus (DG) and cornu ammonis 2 (CA2) field by P7. Expression in these regions then becomes strong and stable in adults. (**B**) pSmad/BRE-gal IHC of adult sagittal sections. Like BRE-gal, pSmad is expressed in the DG and CA2 regions, but is also expressed in CA3 and CA1. In the DG, virtually all BRE-gal-expressing cells also label for pSmad (right panel). However, some pSmad-positive cells in subgranular and supragranular zones of the DG are BRE-gal-negative (inset; arrows). (**C**) IHC of adult sagittal sections, confocal images. Nearly all Prox1-expressing DG granule neurons are BRE-gal positive. A few BRE-gal+/Prox1- cells are also observed (arrowheads). BRE-gal is not expressed by Sox2-positive neural stem cells of the subgranular zone (SGZ), although coexpression is seen in some hilar cells (asterisk). (**D**) IHC of adult sagittal sections, confocal images. Lhx2 is expressed in a subset of Sox2-positive neural stem cells (arrowheads in right panel), but not in BRE-gal-positive granule neurons. Scale bars: 200 um (A,B), 50 um (inset in B,C,D). Abbr: DG, dentate gyrus; CA, cornu ammonis; cpe, choroid plexus epithelium; hcf, hippocampal fissure.

Away from the DTM, BRE-gal expression in whole mount embryos was evident in scattered cells at E11.5 and E12.5 (Fig. 1E,F). Coronal sections revealed reporter-positive cells throughout the E11.5–E17.5 period in the cortical marginal zone (Figs. 1H–I, 2B, S1A), where hem-derived neurons, including Cajal-Retzius neurons (CRN), are known to migrate and differentiate [21]. Consistent with a hem origin, labeled marginal zone cells were detected farther away from the hem with increasing age [22] (compare Figs. 1H–I, 2B, and S1A), and two-color fluorescent immunohistochemistry (IHC) with the neuron-specific marker TuJ1 suggested that all BRE-gal-positive cells are neurons (Fig. S1B). Not all TuJ1-positive cells in the marginal zone expressed BRE-gal, which is consistent with the cortical hem being the source for only a subset of marginal zone neurons [23]. In adult mice, BRE-gal positive cells were also found in the marginal zone of the hippocampus (Fig. 3A) and neocortex (data not shown), where CRNs are known to persist [24], [25].

LacZ is well known for its perdurance, but perdurance alone seemed unlikely to account for BRE-gal expression in marginal zone neurons at such late embryonic and adult stages. Rather, this suggested ongoing BMP signaling in marginal zone neurons. To test for this, we performed double labeling studies for pSmad and lacZ. Essentially every BRE-gal-positive cell in the marginal zone showed high nuclear pSmad immunoreactivity (arrowheads in Fig. S1C), indicating ongoing Bmp signaling in these cells.

### The BRE-gal Expression Border Coincides with the Cortex-hem Boundary

With the exception of the marginal zone, BRE-gal expression was not seen in the cortical neuroepithelium, suggesting that the BRE-gal expression border approximates the cortex-hem boundary (CHB). To examine this more closely, we first compared BRE-gal to Lmx1a, which is selectively expressed by hem cells, but not cortical radial glia [26]. Two-color fluorescent IHC demonstrated that BRE-gal and Lmx1a largely coincide (Fig. 1J–L), although as expected for an independent signaling reporter, BRE-gal expression did not coincide precisely with Lmx1a (Fig. 1K). We then compared BRE-gal to Lhx2, the cortical selector gene that suppresses hem fate and therefore defines the CHB [16]. BRE-gal and Lhx2 expression negatively correlated and were closely apposed, with relatively few BRE-gal-expressing cells in the Lhx2 cortical domain (Fig. 1L). This indicates that the BRE-gal expression border corresponds closely to the CHB.

### BRE-gal is Restricted to Neural Tissues with High Bmp Signaling: Comparisons to Nuclear pSmad and Msx1 Readouts

Expression of BRE-gal in the DTM is consistent with the high Bmp signaling that is known to occur there [7], [9], [11], [12]. To assess this in further detail, we compared BRE-gal expression to pSmad at E10.5 and E12.5. As demonstrated previously [11], [12], nuclear pSmad immunoreactivity at E10.5 was graded within the dorsal telencephalon, with highest levels in the DTM (Fig. 2A). By E12.5, overall pSmad signal intensity was lower, although the spatial gradient was maintained (Fig. 2B). Strong pSmad immunoreactivity was also seen at the ventricular surface, consistent with previous studies (Fig. 2B) [11], [27]. At both E10.5 and E12.5, significant pSmad immunoreactivity was present in the head mesenchyme, where BRE-gal was not expressed (Fig 2A–B, arrows). BRE-gal expression therefore occurred in neural tissues with high pSmad levels, but not in non-neural tissues with significant nuclear pSmad expression.

The relatively sharp border of BRE-gal expression did not correlate linearly with the graded distribution of nuclear pSmad. We therefore compared BRE-gal to Msx1, an evolutionarily-conserved positional determinant of high Bmp signaling [2], [28] that also exhibits a sharp expression border in the DTM [9], [12]. Using Msx1-nlacZ mice, which utilizes the same nuclear lacZ reporter present in the BRE-gal mice [29], we observed Msx1-nlacZ localization to the DTM at E10.5 and E12.5, but its expression did not extend as far laterally as BRE-gal (Fig. 2A,B). In addition, Msx1-nlacZ was expressed in head mesenchyme, unlike BRE-gal (arrows in Fig. 2A,B). Thus, BRE-gal is expressed in DTM cells that have high nuclear pSmad levels and in a larger domain than Msx1, but is not expressed by all cells with high pSmad and Msx1, such as head mesenchymal cells.

### BRE-gal Expression is Activated and Maintained in the Hippocampus

Although BRE-gal was not expressed in the embryonic hippocampal anlagen (Figs. 1G–I and Fig. S1A), expression became detectable in the hippocampus of postnatal animals and was strong in adults (Fig. 3A). In contrast, Xgal staining of CPE cells in the lateral ventricles, which was initially strong and uniform at embryonic stages, became mosaic in adults. Within the adult hippocampus, expression was seen in the dentate gyrus (DG) and cornu ammonis (CA) fields. BRE-gal expression was particularly strong in CA2 and the dentate gyrus (DG), with more scattered expression in CA1 (Fig. 3A). IHC with antibodies against bacterial lacZ confirmed the strong expression of the BRE-gal reporter in the adult hippocampus and CPE (Fig. 3B; data not shown).

**Figure 4:**
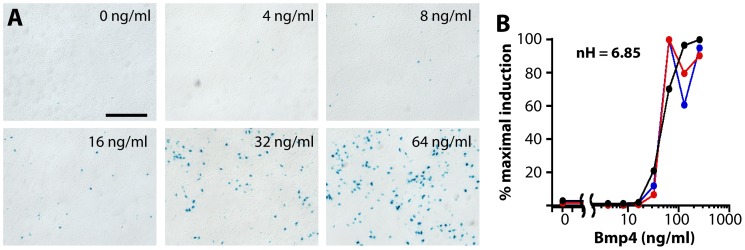
Ultrasensitive BRE-gal induction by Bmp4 *in vitro*. (**A**) Xgal stains, dissociated E12.5 cortical cells. BRE-gal expression displays a threshold effect, with strong activation occurring between 16–32 ng/ml. At all concentrations, BRE-gal induction is highly mosaic. Scale bar: 200 um. (**B**) RT-qPCR, dissociated E12.5 cortical cells. BRE-gal activation is consistent and highly sigmoidal (three independent cultures graphed separately), with an EC50 of 42.23 ng/ml (+/−0.15) and a Hill coefficient of 6.85 (+/−0.08).

BRE-gal expression in the adult DG was uniform, and IHC studies indicated that essentially all Prox1-positive DG granule neurons also expressed BRE-gal (Fig. 3C). Conversely, most BRE-gal-expressing cells in the DG were Prox1-positive, although scattered BRE-gal-positive, Prox1-negative cells were also present (Fig. 3C). The DG also contains Sox2-expressing neural stem cells in the subgranular zone (SGZ), a well-known stem cell niche of the adult brain [30], [31], [32]. Co-labeling revealed that BRE-gal expression was absent from Sox2-positive cells (Fig. 3C). Allen Brain Atlas images and our own *in situ* hybridizations indicated that Lhx2 is also expressed in the subgranular zone of adult hippocampus (Fig. 4C; data not shown), and IHC studies revealed that Lhx2 is selectively expressed by a subset of Sox2-positive DG progenitors (Fig. 3D). Accordingly, like Sox2 and BRE-gal, Lhx2 and BRE-gal expression were mutually exclusive (Fig. 3D). Thus, essentially all DG neurons express BRE-gal in a strong and non-mosaic fashion, while DG progenitors do not express BRE-gal.

We then performed two-color fluorescent IHC for BRE-gal and pSmad in adult hippocampal sections. Nuclear pSmad was detected in all hippocampal regions, particularly in CA3, the DG hilus, and subgranular zone (Fig. 3B). Similar to the embryonic telencephalon, only a subset of hippocampal cells with abundant pSmad expressed BRE-gal (Fig. 3B; inset; arrows). Conversely, essentially all BRE-gal positive cells were strongly pSmad immunoreactive. Collectively, these studies revealed that: 1) BRE-gal expression in the hippocampus is initiated after the embryonic period, culminating in particularly strong expression in the DG and CA2, 2) BRE-gal is expressed by essentially all DG granule neurons, 3) BRE-gal expression coincides with high nuclear pSmad levels, suggesting ongoing Bmp signaling in DG neurons, and 4) similar to the embryonic telencephalon, BRE-gal is expressed in some, but not all, hippocampal cells with abundant pSmad.

### BRE-gal Activation by Bmp4 is Ultrasensitive in Cortical Precursor Cells

The selective responsivity of BRE-gal to Bmps, but not to other families of signaling molecules, was established in the mouse ES cell line used to generate BRE-gal mice (see accompanying manuscript). To test for the Bmp responsivity of BRE-gal in telencephalic cells, we added human recombinant Bmp4 to dissociated E12.5 cortical precursor cells (CPCs), which endogenously do not express BRE-gal (Figs. 1,2). Little to no BRE-gal expression was observed in CPCs after 3 days *in vitro* without Bmp4. Addition of Bmp4 (4–64 ng/ml) after the first day in culture led to dose-dependent BRE-gal activation (Fig. 4A). Activation was particularly dramatic between 16 and 64 ng/ml Bmp4, suggesting a threshold effect for BRE-gal induction. In addition, BRE-gal expression was highly mosaic at all concentrations tested (Fig. 4A).

The strong threshold effect and mosaicism of BRE-gal activation in Bmp4-treated CPCs were highly reminiscent of Msx1 activation in these cells, which occurs via a cell-intrinsic ultrasensitivity mechanism [12]. Ultrasensitivity refers to switch-like, “all-or-none” phenomena characterized by sigmoidal dose-response curves and Hill numbers (nH) greater than 1 [33]. (Note: “Ultrasensitivity” is an established term that specifically refers to switch-like threshold responses to a stimulus rather than extreme sensitivity to low stimulus concentrations.) In our system, CPC ultrasensitivity to Bmp signaling has been implicated in DTM fate induction and the initial sharpening of its border with the cortical primordium [12]. To determine whether BRE-gal activation is also ultrasensitive to Bmp4, we quantified BRE-gal levels in CPCs cultured across a range of Bmp4 concentrations by RT-qPCR. This yielded a highly sigmoidal BRE-gal dose-response curve (nH = 6.85+/−0.08) with the largest changes in expression occurring between 32 and 64 ng/ml Bmp4 (EC50 = 42.23+/−0.15) (Fig. 4B). For comparison, Msx1 in similar cultures was less ultrasensitive (nH = 2.4–3.8), while the Bmp4-responsive gene Tgif [11] was not ultrasensitive (nH = 0.3) [12]. These studies indicate that BRE-gal activation by Bmp4 in dissociated CPCs is highly ultrasensitive.

We also examined BRE-gal responsivity to Bmp4-soaked beads in E10.5 telencephalic explants. Although some BRE-gal activation occurred in control explants independent of beads or Bmp4 (data not shown), blinded scoring by multiple individuals revealed that Bmp4-soaked beads invariably elicited more BRE-gal expression than BSA beads within the same explants (Fig. S3; n = 5 explants; see Materials and Methods). Like its activation in dissociated CPCs, BRE-gal activation around Bmp4-soaked beads was also mosaic. These observations in explants confirmed the responsivity of BRE-gal to Bmp4 and the ultrasensitive nature of BRE-gal activation.

### High Bmp Signaling is not Required for Hem Differentiation

**Figure 5:**
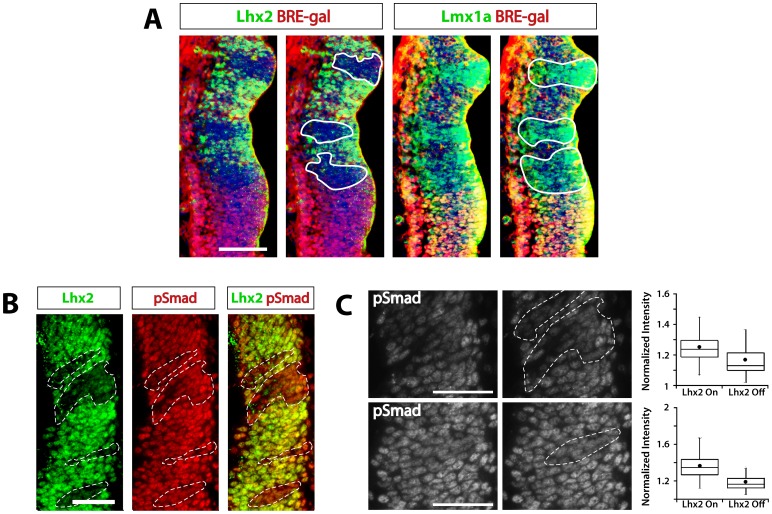
Absence of BRE-gal or increased pSmad levels in ectopic hem cells. IHC of coronal sections from E12.5 Lhx2 null chimeras, confocal images. (**A**) The normal hem (towards bottom of panels) expresses BRE-gal and Lmx1a, but not Lhx2. Lhx2 null patches (white lines) seen in adjacent sections of the medial pallium upregulate Lmx1a, but do not express BRE-gal. Scale bar: 100 um. (**B**) pSmad levels (red) are not elevated in Lhx2 null patches in the medial pallium (white dashed lines). Scale bar: 50 um. (**C**) Monochrome pSmad images shown in (B) and boxplots of their relative pSmad intensities (normalized to Hoechst intensity). pSmad intensity is lower in “Lhx2-off” (Lhx2 null) hem cells compared to adjacent “Lhx2-on” medial pallial progenitors (p<0.0001 for both sections). Non-normalized pSmad intensity values were also statistically significant (p = 0.007 for patches away from endogenous hem (top of panel), p = 0.0001 for patches closer to hem (bottom of panel)). Scale bar: 50 um.

The restriction of BRE-gal to the cortical hem provided an opportunity to study Bmp signaling in ectopic hem cells of Lhx2-null chimeras [16]. In these chimeras, Lhx2-null cells form “patches” in the medial pallium, where BRE-gal is not expressed, which transform into functional hem cells. If high Bmp signaling were required for this transformation, then the ectopic hem cells should express BRE-gal and higher nuclear pSmad levels than surrounding wild-type (Lhx2-positive) cells. However, while medial Lhx2-null patches ectopically expressed the hem marker Lmx1a, they did not express BRE-gal (Fig. 5B). We also failed to detect ectopic BRE-gal expression in adjacent sections by Xgal staining, which is more sensitive than IHC (data not shown). Furthermore, the ectopic hem cells did not have higher nuclear pSmad levels than adjacent cortical cells (Fig. 5C). In fact, intensity measurements suggested reduced pSmad levels in the ectopic hem cells compared to their Lhx2-positive neighbors (p<0.001 for each of two Lhx2-null patches; Fig. 5C). Thus, unlike normal hem cells, ectopic hem cells in Lhx2 null chimeras do not express BRE-gal or high pSmad levels. This indicates that hem fate in the medial pallium can be specified with Bmp signaling intensities that are lower than those seen in the normal hem, if Lhx2 is absent.

## Discussion

In this report, we characterized BRE-gal mice, a novel Bmp activity reporter, and used these mice to define new cell types with high-level Bmp signaling and to address mechanisms in cortical hem differentiation. BRE-gal activity in telencephalic cells *in vivo* and *in vitro* is restricted to cells with high Bmp signaling, but high-level signaling alone is not sufficient to activate BRE-gal. At embryonic stages, BRE-gal expression is prominent in the DTM and in marginal zone neurons, then at later stages in DG granule neurons and certain pyramidal neurons of the hippocampus. Both *in vivo* and *in vitro*, BRE-gal activation displays ultrasensitivity, and its sharp expression border *in vivo* defines the CHB with remarkable precision. However, ectopic Lhx2-null hem cells in the medial pallium do not express BRE-gal or increased pSmad levels, which has multiple implications for the mechanisms involved in hem development (see below).

### BRE-gal Comparison to Other Bmp Reporters and Readouts

Three previous Bmp reporter mouse lines have been described [4], [5], [6], which all utilized a BRE from the mouse *Id1* enhancer [34]. Like the *XId3* BRE in our BRE-gal mice, the *mId1* BRE is an “SMM”-type BRE – i.e. it has Smad1 and Smad4 binding sites separated by five nucleotides (Fig. 1A) [18], [34]. (“SMM” stands for schnurri-mad-medea, with mad and medea being the *Drosophila* counterparts of Smad1 and Smad4, respectively.) However, the *mId1*-based reporter constructs also included a second Bmp responsive element (GGCGCC) that is required for Bmp responsivity [34], whereas the *XId3* BRE alone is sufficient for Bmp-responsive transcriptional activity [18]. Telencephalic expression was not evident in two of the *mId1*-based mouse lines [5], [6], but was seen in the line generated by Blank et al. [4]. Like our BRE-gal line, this “BRE-lacZ” line was generated via blastocyst injection of stable mouse ESC lines screened for Bmp responsivity. Two BRE-lacZ lines with independent integration sites were described, and while the telencephalon was not described in detail, these two lines appeared to display differences in dorsal telencephalic expression – one was expressed in head mesenchyme and in cortex proper, but not in the hem or CPE (although staining of choroid plexus mesenchyme may be present), while the other showed restricted expression in the marginal zone (see supplement for Blank et al. [4]). The second pattern, but not the first, resembles our BRE-gal line (Fig. 2B). The neural specificity of BRE-gal should be a useful feature for future studies of Bmp signaling in neural tissues. Although the reasons for this specificity are uncertain, our studies rule out integration site or the *XId3* promoter as explanations.

The fact that BRE-gal is not expressed in all cells with high BMP activity is not entirely surprising. As discussed earlier, at least two BRE-lacZ lines did not express well, if at all, in developing forebrain. The lack of correspondence is not unique to BMP signaling reporters. For example, three well known reporters of canonical Wnt signaling (BATGAL, TOPGAL, and Axin2^lacZ^) – which include two based on the same Lef/TCF binding site (BATGAL and TOPGAL) – exhibit significant spatial and temporal differences in expression within the same organ (lung) in both normal and injured contexts [35]. This speaks to the importance of context on multiple levels, including developmental and genomic (e.g. integration site). Thus, it is not uncommon – and perhaps should be expected – that individual signaling reporter lines will display unique features, highlighting the need for different Bmp reporter lines. It is important to emphasize that while BRE-gal is not expressed everywhere high pSmad is present, pSmad was detected in all tissues and cells expressing BRE-gal. BRE-gal therefore acts much like other well known BMP target genes, such as the *Msx* and *Id* genes, which are expressed in some, but not all cells with high BMP signaling.

Our BRE-gal line therefore has unique features that should prove useful for BMP signaling studies by others. In particular, the BRE-gal line provides for the analysis of a BRE that is distinct from the one used in previous BRE-lacZ lines, enables studies on BMP signaling in the developing brain that are selective to neuroepithelial-derived cells, and provides the opportunity to dissect the molecular and transcriptional mechanisms underlying ultrasensitivity. Other advantages of the BRE-gal line outside of the CNS are described in the companion manuscript.

### Bmp Signaling in Neurons of the Marginal Zone and Dentate Gyrus

The BRE-gal and pSmad studies revealed ongoing Bmp activity in two neuronal subtypes where Bmp signaling has not been well-studied – cortical marginal zone neurons, which likely include Cajal-Retzius neurons, and DG granule neurons. Bmp signaling has an established role in maintaining the quiescence of neural stem cells in the DG subgranular zone [36], [37], [38], [39], but BRE-gal is not activated in Lhx2- or Sox2-expressing DG progenitors. This apparent difference between BRE-gal and pSmad in the adult DG appears to represent another example of BRE-gal being expressed in some, but not all, cell types with significant Bmp signaling. Bmps can act as chemorepellents during axon guidance [40], [41], as retrograde signals that negatively regulate axon-target interactions [42], and as enhancers of dendritic growth [43], all of which represent potential roles for Bmp signaling in marginal zone and DG granule neurons.

### BRE-gal as an Ultrasensitive Bmp Signaling Reporter in the Dorsal Telencephalon

Ultrasensitivity is a well known phenomenon that underlies many biochemical and cellular phenomena (e.g. kinase reactions and cell fate specification) [33]. In our system, CPC ultrasensitivity to Bmps has been implicated as a mechanism for initial induction and specification of DTM cell fates and their borders *in vivo* [12]. In these earlier studies, CPC ultrasensitivity was described at the level of Msx1 expression. Like Msx1, BRE-gal expression in cultured CPCs is ultrasensitive to Bmp4 (Fig. 4B), and BRE-gal ultrasensitivity *in vitro* correlates with its sharp expression border *in vivo* (Fig. 1J). The relatively small and defined nature of BRE-gal regulatory sequences (compared to the *Msx1* gene) should help to decipher the molecular mechanisms underlying ultrasensitivity to Bmp, which remain unknown. It is important to note, however, that the multimerization of BRE sites in the BRE-gal reporter construct could itself lead to ultrasensitivity due to cooperative transcription factor binding [44], [45], [46]. That being said, BRE-gal expression in the diencephalic dorsal midline appears graded rather than sharply bordered (data not shown), which suggests that BRE multimerization may not be sufficient for ultrasensitivity *in vivo*. Interestingly, Msx1 expression is also graded in the diencephalon (data not shown), which raises the possibility that similar mechanisms may regulate the sharpness of BRE-gal and Msx1 expression borders. This also suggests that the BRE-gal reporter may serve as a more graded readout of BMP signaling in diencephalic cells, although additional studies on this point will be needed to complement the *in vivo* observations.

### Bmp Signaling in Cortical Hem Differentiation

The cortical hem is a hippocampal organizer [16], and previous studies have implicated both high-level Bmp signaling and Lhx2 absence in its induction [10], [11], [13], [16]. In the normal hem primordium, Bmp signaling is high (Fig. 2A and B) [11], Lhx2 expression is low (Fig. 1J) [10], [16], and explant and genetic studies suggest that high Bmp signaling suppresses Lhx2 [10], [11]. In the medial pallium – where Lhx2 expression is high and Bmp signaling is lower than in the normal hem – constitutive or mosaic Lhx2 inactivation leads to cell-autonomous transformation of cortical neuroepithelial cells into hem cells [10], [16], [17]. In the current study, we found that the Lhx2-null hem cells in medial pallium express neither BRE-gal nor high pSmad levels (Fig. 5A–C). In fact, pSmad levels appeared lower in the ectopic hem cells than in their immediate neighbors (Fig. 5C). Thus, Lhx2-null medial pallial cells do not require high-level Bmp signaling for hem differentiation.

These findings indicate that hem differentiation can occur in either of two scenarios: 1) with high Bmp signaling and Lhx2 suppression, as occurs in the normal hem, or 2) with lower Bmp signaling levels and forced Lhx2 inactivation in the medial pallium. In turn, these two scenarios suggest that hem fate can be specified with different Bmp signaling intensities depending on Lhx2 status, including lower intensities that are insufficient to activate BRE-gal. In addition to having sufficient Bmp signaling, hem specification may also require Lhx2 suppression or absence, although Lhx2 misexpression studies in the normal hem anlagen will be needed to formally address this requirement.

### Lhx2 and Bmp Signaling in the Medial Pallium and Dentate Gyrus

Our findings further indicate that Lhx2 cortical selector activity does not involve the regulation of Bmp signaling intensity. Previous Lhx2 null studies [16] were potentially consistent with Lhx2 suppression of Bmp signaling intensity as a potential mechanism underlying Lhx2-mediated specification of cortical identity and suppression of hem fate. However, ectopic Lhx2-null hem cells expressed neither BRE-gal nor higher pSmad levels, which would be expected if this model were correct. Moreover, Lhx2 and pSmad expression patterns do not correlate across the normal cortex-hem boundary (CHB), since pSmad levels are smoothly and continuously graded across the CHB [11] defined by the sharp Lhx2 expression border [16]. Taken together, these findings indicate that Lhx2 does not have a primary role in regulating Bmp signaling intensity in neuroepithelial cells, but instead regulates how these cells interpret Bmp signaling.

The Lhx2, BRE-gal, and pSmad findings in adult hippocampus suggest that a similar relationship between Lhx2 and Bmp signaling may exist in the adult DG. As in the embryonic medial pallium, Lhx2 and BRE-gal expression in the adult DG have a relatively precise negative relationship (Fig. 3D). Since the DG arises from the embryonic CHB region, these similarities are perhaps unsurprising. Nonetheless, they suggest that like cells of the embryonic medial pallium, Lhx2 does not primarily regulate Bmp signaling intensity in adult DG cells, but instead is likely to regulate how DG cells respond to Bmp signals. The selective expression of Lhx2 in a subset of DG progenitors (Fig. 3D) could also account for why Bmp signaling is required to maintain the quiescence of some DG stem cells, but not others [37].

## Materials and Methods

### Generation of Transgenic BRE-gal Lines

The generation of the construct and BRE-gal mouse lines are described in greater detail in the companion manuscript by Javier et al. To generate the BRE-gal construct, a 7xBRE-GFP construct [18] was cut with HindIII and SpeI to remove the GFP portion. The nuclear LacZ (nLacZ) construct from the Stefano Piccolo lab [47] was cut with HindIII/XbaI/ScaI and ligated with the 7xBRE. The 7xBRE-nLacZ construct was linearized with XmnI. Pronuclear-injected mouse lines were generated by injection of linearized BRE-gal construct into CB6F1 pronuclei (NCI). Nine transgenics were identified by PCR genotyping for *LacZ* (see below), and backcrossed with B6 mice to generate F1 mice. We screened for germline transmission of the transgene by PCR genotyping of the F1 progeny, and identified two founders from which we established two separate pronuclear-injected BRE-gal lines. The transgenic mice grow to full adulthood without any noted abnormalities. To generate the mESC-derived line, the linearized BRE-gal construct was electroporated into 129P2/Ola derived E14 embryonic stem (ES) cells (UCI Transgenic Mouse Facility). Clonal lines were screened for responsivity and specificity to Bmp *in vitro* (see accompanying manuscript) and for euploidy (>80% normal karyotype). A mouse line carrying the BRE-gal construct was then obtained by aggregating recombinant ES cells of the selected clonal line with single 8–16 cell embryos from C57BL/6N mice (Taconic). Nine chimeric founder mice were identified based upon coat color. Founders were screened for germline transmission by PCR genotyping of F1 progeny (generated by backcrossing with CD1). Two founders were capable of germline transmission. F2 embryos from both founders (generated by backcrossing F1 with CD1) were evaluated by Xgal wholemount and histochemistry to determine that both founders generated BRE-gal mice with similar patterns and strengths of expression. The transgenic mice grow to full adulthood without any noted abnormalities. All subsequent analysis in the article used F3 progeny from only one line.

### Mouse Matings, Genotyping, and Tamoxifen Studies

Noon of the vaginal plug day was designated day 0.5, and developmental stages were confirmed by crown-rump measurements. The following mice and matings were used: BRE-gal (homozygotes or hemizygotes; 75% CD1, 25% 129 background) X CD1 (Charles River, Wilmington, MA) for BRE-gal studies; Msx1-nlacZ (heterozygotes; C57BL/6J background) [48] X CD1 for Msx1 studies; CD1 intercrosses for wild-type studies; Lhx2^cKO/cKO^/BREgal X R26^CreER/CreER^;Lhx2^sKO/+^ (R26^CreER^, JAX stock 004847 [49]; Lhx2 sKO allele, C57BL/6J background, [50]) for Lhx2 null chimera studies. Genotypes were determined by Xgal staining of bodies or tissues (BRE-gal) and/or PCR genotyping of tail DNA using previously described methods and primers [16], [47]. Tamoxifen (Sigma, St. Louis, MO) at 5 ug/g body weight was injected into timed-pregnant females at E5.5, and Lhx2 null chimeras were harvested, as described previously [16].

### Histochemistry, Immunohistochemistry, and *in situ* Hybridization

These were performed on 20 um cryosections or cultured cells, and imaged as previously described [10], [12], [16] using an *Id3* probe (Robert Benezra) and the following primary antibodies: pSmad1/5/8 (rabbit polyclonal against human Smad5 phosphopeptide, which recognizes dual phosphorylated Smad1, Smad5, and Smad8; 1∶80; Chemicon AB3848, Temecula, CA), lacZ (mouse monoclonal; 1∶12,500; Developmental Studies Hybridoma Bank 40–1a, Iowa City, IA), Lhx2 (rabbit polyclonal; 1∶100; [16]), Lhx2 (goat polyclonal; 1∶100; Santa Cruz Biotechnology sc-19342, Santa Cruz, CA), Prox1 (mouse monoclonal; 1∶250; Chemicon MAB5652), Sox2 (goat polyclonal; 1∶200; Santa Cruz Biotechonology sc-17320), Lmx1a (rabbit polyclonal; 1∶1000; courtesy of Michael German, UCSF), TuJ1 (rabbit polyclonal; 1∶2500 dilution; Research Diagnostics RDI-tubuB1abR, Concord, MA). Secondary antibodies were Alexa 488-conjugated goat anti-rabbit, Alexa 555-conjugated donkey anti-goat, and Alexa 555-conjugated goat anti-mouse (1∶200 dilution; Invitrogen A11034, A21432, and A21424, Carlsbad, CA). Hoechst 33342 (Invitrogen, Carlsbad, CA) or eosin (Fisher Scientific) were used for counterstaining. Xgal stains of embryos or explants were performed as described [9] with the following modifications: tissues were fixed for 30–60 minutes in 4% paraformaldehyde supplemented with 2 mM MgCl2 and 5 mM EGTA, then stained in the dark at room temperature for 1 hour. Because endogenous beta-galactosidase activity in adult CPE and CA regions of hippocampus can sometimes be detected by Xgal stains, adult BRE-gal-positive sections were stained in parallel with BRE-gal-negative sections, which did not reveal significant Xgal staining.

### Explant Cultures and Scoring

Whole dorsal forebrain explants were prepared ventricular surface up and processed as described [12]. Four hours after plating on Whatman nucleopore membranes (Clifton, NJ), Affigel blue gel beads (BioRad, Hercules, CA) or heparin acrylic beads (Sigma, St. Louis, MO) soaked in 10 ul of 25–50 ug/ml recombinant human Bmp4 (R&D Systems, Minneapolis, MN) or bovine serum albumin (BSA) were placed onto explants using pulled and flame-polished microcapillary pipettes and a mouth aspirator. Beads were rinsed briefly in PBS prior to placement. For each explant, Bmp4 beads were placed on one hemisphere, while control BSA beads were placed on the other. Explants were processed 24 hours after bead placement for whole mount Xgal staining. Explants were blindly scored by 3 individuals for the hemisphere with more Xgal staining surrounding beads; scoring was 100% concordant with no interobserver variability.

### Dissociated Cortical Progenitor Cultures

E10.5 or E12.5 embryonic cortical dissociates were prepared as described [11]. Briefly, skin and mesenchymal layers were manually removed, then cortices were isolated using forceps or microscissors. Cortices were trypsinized with 0.05% trypsin/0.02% EDTA/0.2% BSA in HBSS for 20 minutes at 37C, treated with an equal volume of 1 mg/ml soybean trypsin inhibitor (Sigma) in HBSS, then triturated with P200 pipette tips. Cells were washed with 0.2% BSA in HBSS, then plated at 50,000 cells/ml on laminin-coated coverslips in media containing 20 ng/ml EGF, 10 ng/ml FGF2 (R&D Systems or PeproTech, Rocky Hill, NJ), and 2 ug/ml heparin (Sigma). Bmp4 was added 24 hours after initial plating. RNA was harvested and column purified (BioRad, Hercules, CA) 48 hours following Bmp4 addition.

### Real-time RT-qPCR

RNA preps, cDNA syntheses, PCR quality controls, experimental runs, and statistical methods were performed as described [9], [11]. All primers and amplicons were verified by melting curve analysis, agarose gel electrophoresis, and amplification efficiency testing; experimental amplicons were also validated by sequencing. All cDNA samples were validated for RT reaction efficiency and minimal genomic DNA contamination using 18 S primers (cDNA/genomic target ratio >10^5^) and run in duplicate for 40 cycles. Primers for Cyclophilin A (CYPA), one of the two best internal references among five screened on embryonic cortical tissues and cells (Currle 2005), were used for normalization. RT-qPCR data (StepOne Plus, ABI, Carlsbad, CA) were normalized, and −ΔCt and fold induction values (2^−ΔΔCt^) were calculated. Data were then plotted as percent maximal values (100% maximal value designated as the largest fold induction in each experiment) using Kaleidagraph (Synergy Software, Reading, PA) and fitted to a Hill curve using SigmaPlot (Systat Software, San Jose, CA).

### Imaging and Quantification

Imaging was performed as described [16]. Briefly, images were captured on a Spot RT camera (Diagnostic Instruments, Sterling Heights, MI) and SMZ1500 stereodissecting or E600 upright microscopes (Nikon Instruments, Melville, NY) with brightfield, DIC, or fluorescence optics. Whole mount images were captured by DP controller (Olympus, Center Valley, PA) on an Olympus SZX12 upright microscope. Images were processed and compiled using Photoshop. Image adjustments were limited to brightness/contrast, levels, or color balance. All images used in figures for direct visual comparison were taken from comparable rostrocaudal levels, processed in parallel, imaged with identical acquisition settings, and adjusted in parallel in Photoshop. For pSmad intensity measurements, raw 8-bit images were analyzed in ImageJ. Three images were captured for each section analyzed: an Lhx2 antibody-stained image, a pSmad antibody-stained image, and a Hoechst-stained image. Lhx2-positive nuclei were used to demarcate Lhx2-on regions of interest (ROIs); Lhx2/Hoechst overlays were used to demarcate Lhx2-negative nuclei (Lhx2-off ROIs) (Fig. S4). The ROIs were then used on corresponding pSmad images to measure average pSmad signal intensity (range 0–255), which was normalized to Hoechst intensity. T-tests and p-value calculations were performed in Excel. Means and box plots were generated using Kaleidagraph (Synergy Software).

### Ethics Statement

All animal studies were performed in accordance with Institutional Animal Care and Use Committee guidelines via approved IACUC protocol # 2001–2304. All surgeries were performed on euthanized animals with all efforts made to minimize suffering. Animals were euthanized with carbon dioxide from compressed gas canister prior to surgery, with secondary physical method of cervical dislocation to ensure euthanasia.

## Supporting Information

Figure S1 **BRE-gal and pSmad expression in marginal zone neurons. (A)** Xgal stains of E15.5 and E17.5 coronal sections. BRE-gal expression is seen in the cortical marginal zone more laterally with increasing stage (inset, arrowheads; compare to E11.5 and E12.5 in Figs. 1 and 2). BRE-gal is also strongly expressed in the telencephalic and diencephalic choroid plexus epithelium (cpe). **(B)** BRE-gal/TuJ1 IHC of E11.5 coronal sections, confocal images. All BRE-gal-positive nuclei (red) are juxtaposed to cytoplasm with the pan-neuronal marker protein TuJ1 (green). **(C)** BRE-gal/pSmad IHC of E12.5 coronal sections, confocal images. All BRE-gal-positive nuclei (red) also co-label for pSmad (green; white arrowheads). pSmad labeling in these cells is stronger than in adjacent, BRE-gal-negative cells. Non-specific (non-nuclear) signal is seen in the meninges superficial to the pial surface (dashed line). Artefacts of BRE-gal staining are marked with asterisks where lack of Hoechst staining indicates the absence of nuclei. Scale bars: 200 um (A), 25 um (B,C). Abbr: hc, hippocampal anlage; cpe, choroid plexus epithelium.
(TIF)

Figure S2 **Id3 expression is distinct from BRE-gal in embryos and adults.** RNA in situ hybridization (ISH) assays with mouse *Id3* probe. **(A)** In E12.5 coronal sections, *Id3* expression is detected in cortical hem and mesenchyme, but not in choroid plexus epithelium (cpe). This differs from BRE-gal, which is expressed in hem and cpe, but not in the mesenchyme. Scale bar: 200 um. **(B)** Sagittal section of E13.5 forebrain from the Allen Brain Atlas also displays *Id3* expression in cortical hem and mesenchyme, but not in cpe (left, brightfield image; right, expression mask). Scale bar: 2 mm. **(C)** Coronal section of adult forebrain from the Allen Brain Atlas shows only scattered *Id3* expression in neocortex and hippocampus, whereas BRE-gal expression in the hippocampus is strong at this age. Scale bar: 2 mm. Abbreviations: m, mesenchyme, cpe, choroid plexus, h, hem, dg, dentate gyrus.
(TIF)

Figure S3 **Induction of BRE-gal in explants by exogenous Bmp4-soaked beads.** LacZ induction around Bmp4-soaked beads is greater than that around BSA-soaked beads in E10.5 BRE-gal telencephalic explants with blue Affigel beads (n = 5) (A) and E10.5 telencephalic explants from a pronuclear-injected line with clear heparin acrylic beads (n = 2) (B) cultured for 2 days. LacZ induction by exogenous Bmp4 is highly mosaic in the explants from both lines. Scale bars: 0.5 mm (low power) and 0.2 mm (magnified images).
(TIF)

Figure S4 **pSmad intensity in Lhx2-on and Lhx2-off cells.** Raw grayscale images of E12.5 Lhx2-null patches stained for Lhx2, pSmad, and Hoechst (Hoechst not shown). Using ImageJ, nuclear regions of interests (ROIs) were demarcated and categorized as either Lhx2-on or Lhx2-off. pSmad intensities in the ROIs were then measured. All Lhx2-off cells within a section, as shown, were counted in the analysis.
(TIF)
