# Supplementary figures and images for: A Bmp Reporter with Ultrasensitive Characteristics Reveals That High Bmp Signaling Is Not Required for Cortical Hem Fate
Source: PLoS One. 2012 Sep 11;7(9):e44009. doi: 10.1371/journal.pone.0044009 (PMC3439469; doi:10.1371/journal.pone.0044009)

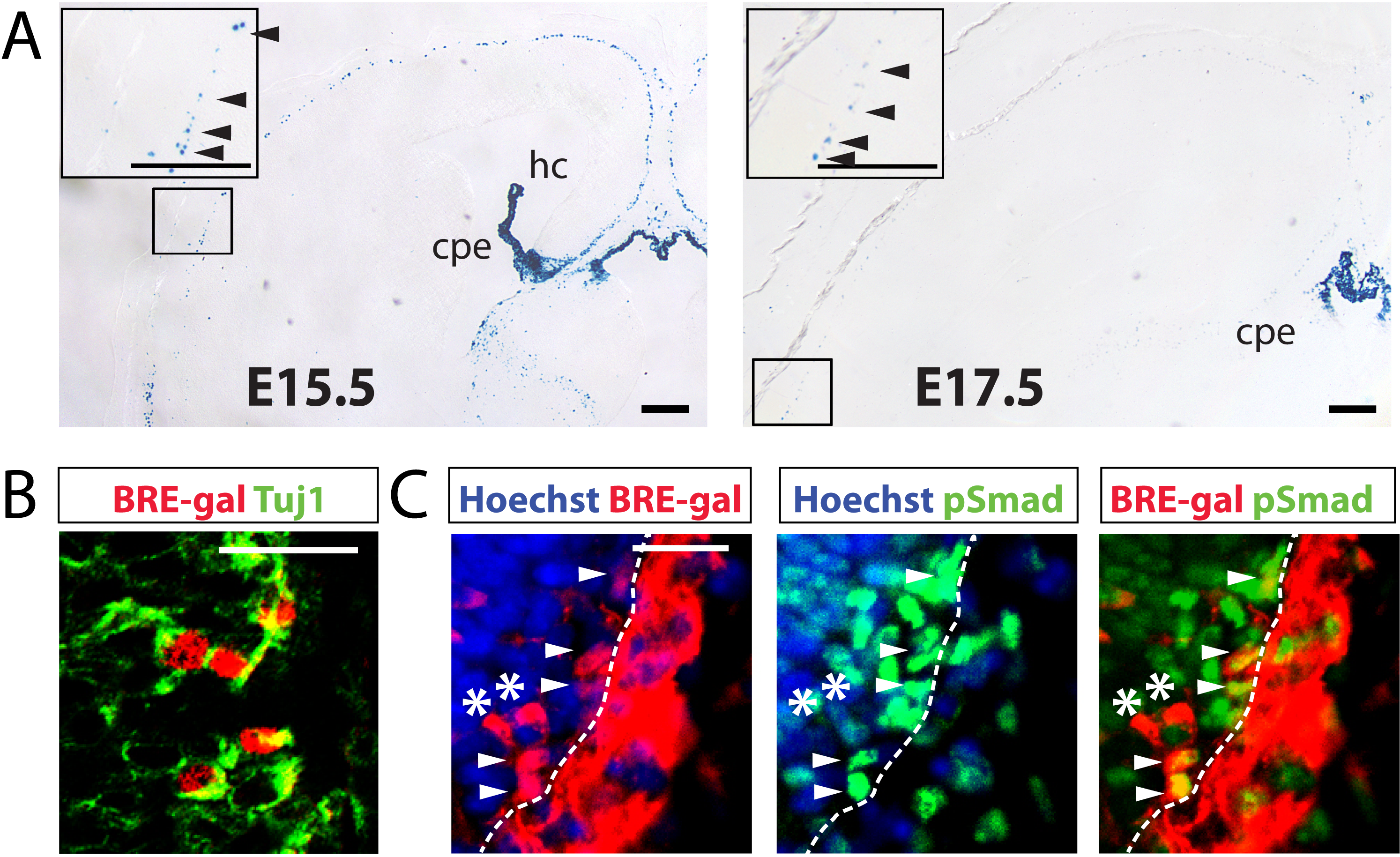

Supplement: Figure S1 — BRE-gal and pSmad expression in marginal zone neurons. (A) Xgal stains of E15.5 and E17.5 coronal sections. BRE-gal expression is seen in the cortical marginal zone more laterally with increasing stage (inset, arrowheads; compare to E11.5 and E12.5 in Figs. 1 and 2). BRE-gal is also strongly expressed in the telencephalic and diencephalic choroid plexus epithelium (cpe). (B) BRE-gal/TuJ1 IHC of E11.5 coronal sections, confocal images. All BRE-gal-positive nuclei (red) are juxtaposed to cytoplasm with the pan-neuronal marker protein TuJ1 (green). (C) BRE-gal/pSmad IHC of E12.5 coronal sections, confocal images. All BRE-gal-positive nuclei (red) also co-label for pSmad (green; white arrowheads). pSmad labeling in these cells is stronger than in adjacent, BRE-gal-negative cells. Non-specific (non-nuclear) signal is seen in the meninges superficial to the pial surface (dashed line). Artefacts of BRE-gal staining are marked with asterisks where lack of Hoechst staining indicates the absence of nuclei. Scale bars: 200 um (A), 25 um (B,C). Abbr: hc, hippocampal anlage; cpe, choroid plexus epithelium. (TIF) [file pone.0044009.s001.tif]

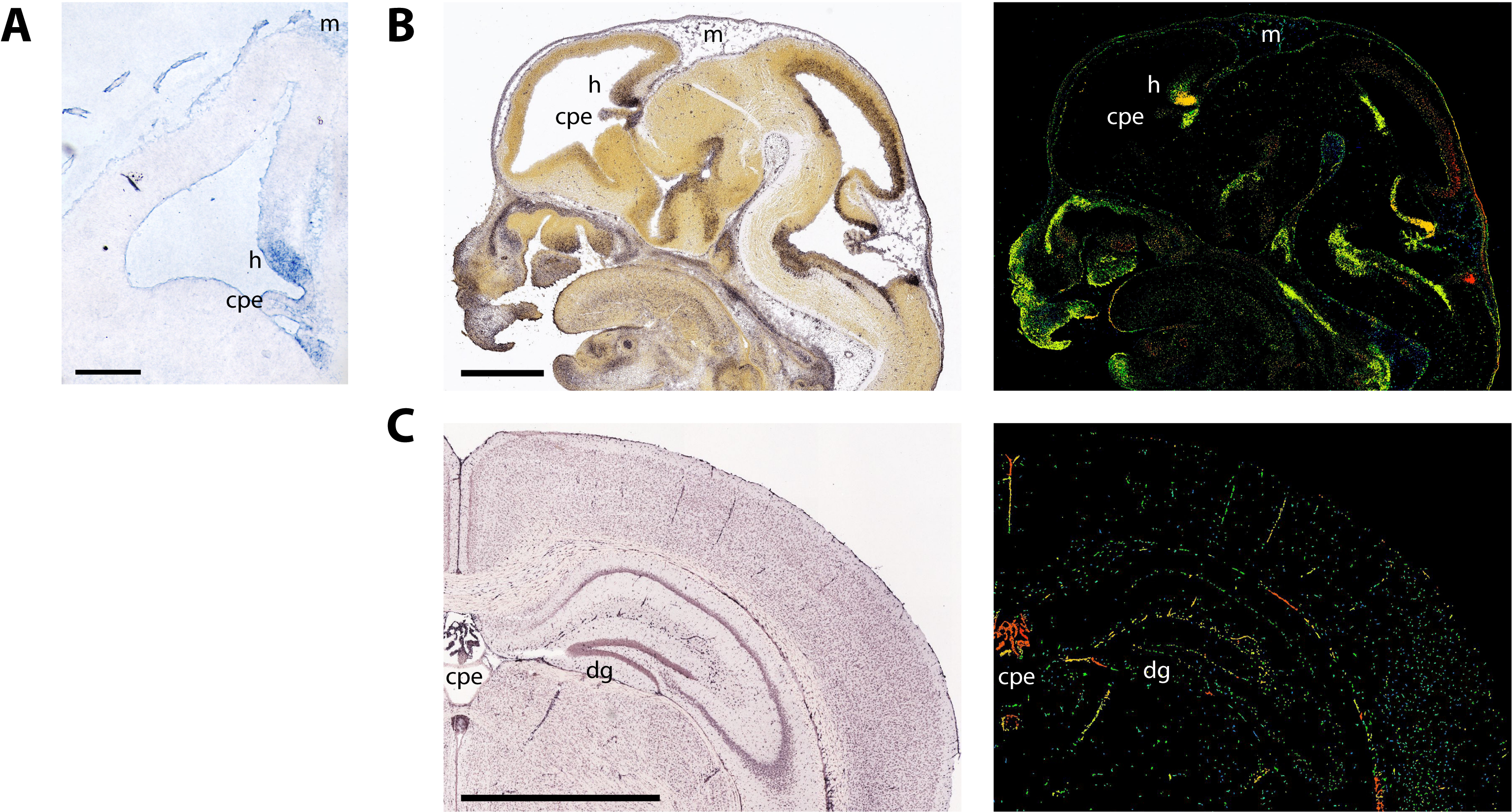

Supplement: Figure S2 — Id3 expression is distinct from BRE-gal in embryos and adults. RNA in situ hybridization (ISH) assays with mouse Id3 probe. (A) In E12.5 coronal sections, Id3 expression is detected in cortical hem and mesenchyme, but not in choroid plexus epithelium (cpe). This differs from BRE-gal, which is expressed in hem and cpe, but not in the mesenchyme. Scale bar: 200 um. (B) Sagittal section of E13.5 forebrain from the Allen Brain Atlas also displays Id3 expression in cortical hem and mesenchyme, but not in cpe (left, brightfield image; right, expression mask). Scale bar: 2 mm. (C) Coronal section of adult forebrain from the Allen Brain Atlas shows only scattered Id3 expression in neocortex and hippocampus, whereas BRE-gal expression in the hippocampus is strong at this age. Scale bar: 2 mm. Abbreviations: m, mesenchyme, cpe, choroid plexus, h, hem, dg, dentate gyrus. (TIF) [file pone.0044009.s002.tif]

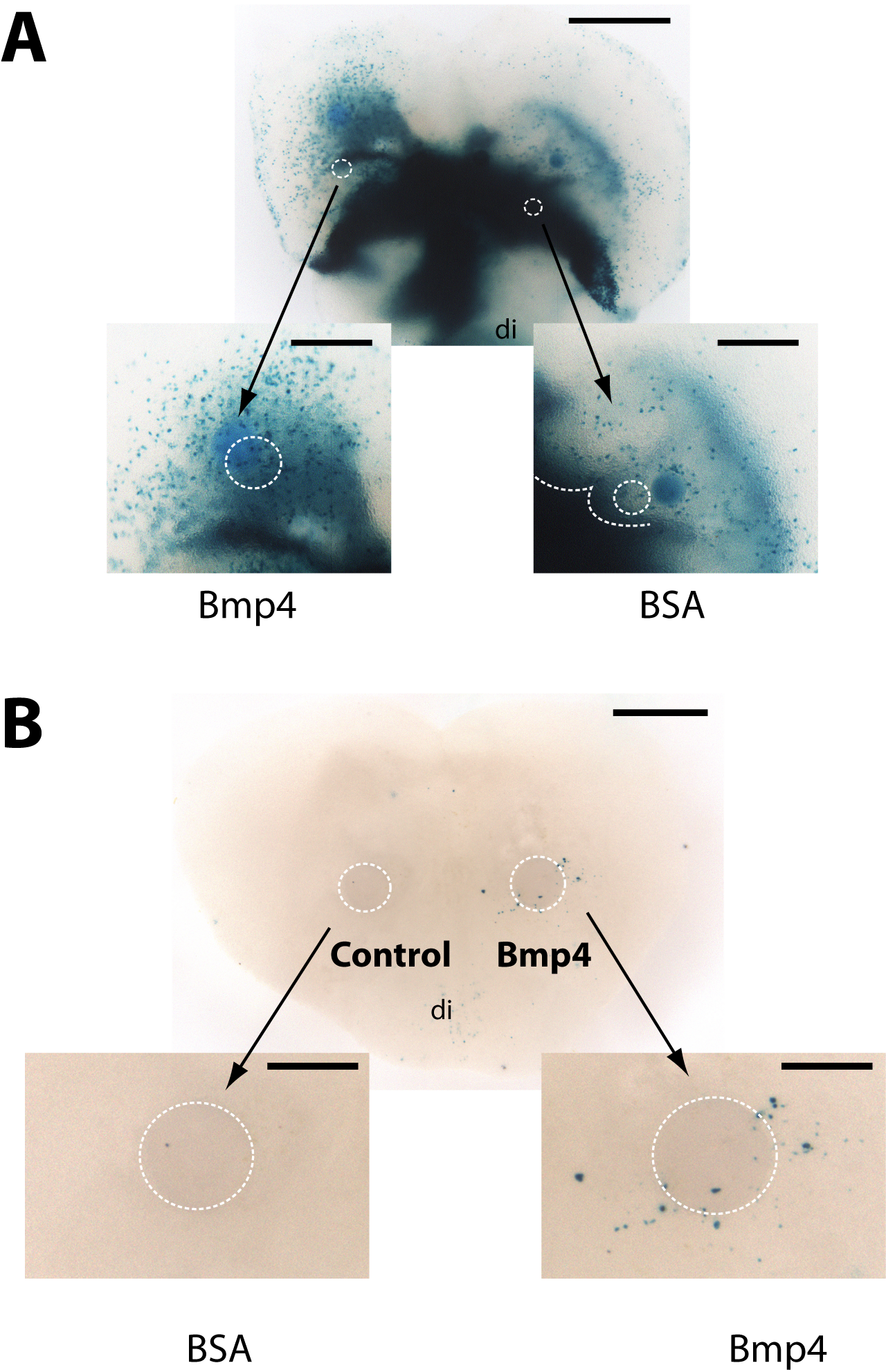

Supplement: Figure S3 — Induction of BRE-gal in explants by exogenous Bmp4-soaked beads. LacZ induction around Bmp4-soaked beads is greater than that around BSA-soaked beads in E10.5 BRE-gal telencephalic explants with blue Affigel beads (n = 5) (A) and E10.5 telencephalic explants from a pronuclear-injected line with clear heparin acrylic beads (n = 2) (B) cultured for 2 days. LacZ induction by exogenous Bmp4 is highly mosaic in the explants from both lines. Scale bars: 0.5 mm (low power) and 0.2 mm (magnified images). (TIF) [file pone.0044009.s003.tif]

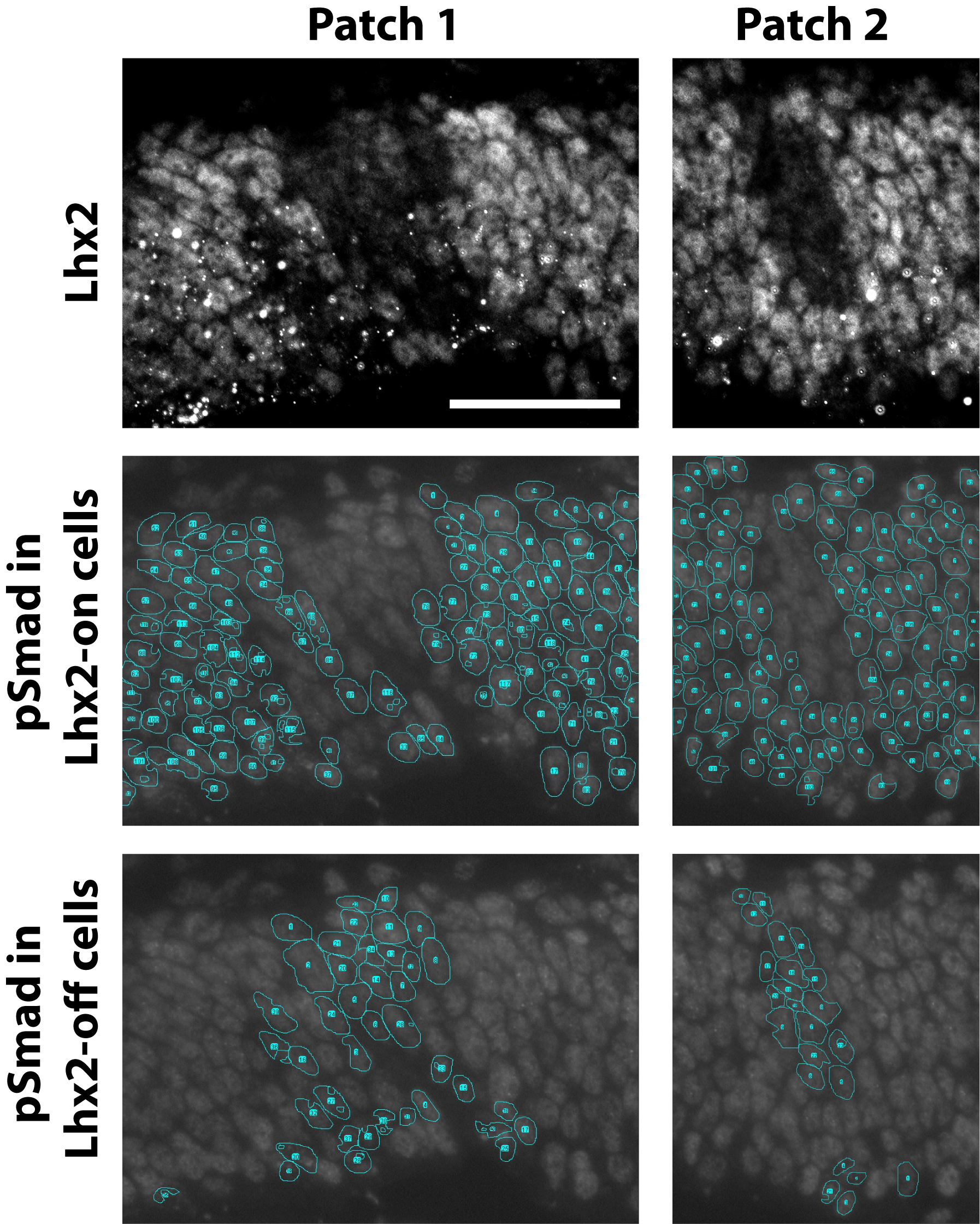

Supplement: Figure S4 — pSmad intensity in Lhx2-on and Lhx2-off cells. Raw grayscale images of E12.5 Lhx2-null patches stained for Lhx2, pSmad, and Hoechst (Hoechst not shown). Using ImageJ, nuclear regions of interests (ROIs) were demarcated and categorized as either Lhx2-on or Lhx2-off. pSmad intensities in the ROIs were then measured. All Lhx2-off cells within a section, as shown, were counted in the analysis. (TIF) [file pone.0044009.s004.tif]
